# QuickStats

**Published:** 2014-10-10

**Authors:** FROM THE NATIONAL CENTER FOR HEALTH STATISTICS

**Figure:**
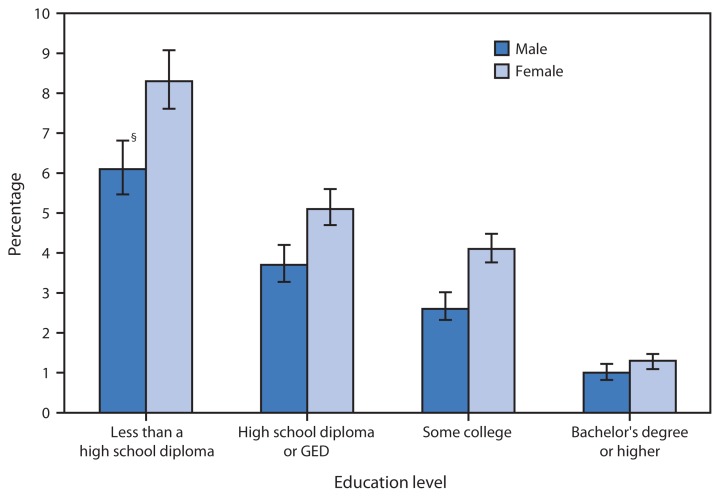
Percentage of Adults Aged ≥25 Years with Serious Psychological Distress,* by Education Level and Sex — National Health Interview Survey,^†^ United States, 2010–2013 **Abbreviation:** GED = general educational development certification. * Serious psychological distress based on responses to the questions, “During the past 30 days, how often did you feel 1) so sad that nothing could cheer you up, 2) nervous, 3) restless or fidgety, 4) hopeless, 5) that everything was an effort, or 6) worthless?” Response codes for the six items for each person were summed to yield a point value on a 0–24 point scale. A value of 13 or more was used to define serious psychological distress. ^†^ Estimates are based on household interviews of a sample of the noninstitutionalized U.S. civilian population. Estimates are age adjusted using the projected 2000 U.S. population as the standard population and using five age groups: 24–44 years, 45–54 years, 55–64 years, 65–74 years, and ≥75 years. ^§^ 95% confidence interval.

During 2010–2013, the total age-adjusted percentage of adults aged ≥25 years with serious psychological distress in the past 30 days was 3.5%. As educational attainment increased, the percentage with serious psychological distress decreased among both men and women. Serious psychological distress was six times higher for adults aged ≥25 years with less than a high school diploma (6.1% of men and 8.3% of women), compared with adults with a bachelor’s degree or higher (1.0% of men and 1.3% of women). At all education levels, women were more likely than men to experience serious psychological distress.

**Source:** National Health Interview Survey. Available at http://www.cdc.gov/nchs/nhis.htm.

**Reported by:** Hashini Khajuria, hwq6@cdc.gov, 301-458-4253; Shilpa Bengeri.

